# Insertion of a pressure sensing arrayminimally affects hindfoot bone kinematics

**DOI:** 10.1186/s13047-015-0081-x

**Published:** 2015-06-24

**Authors:** Tassos Natsakis, Josefien Burg, Greta Dereymaeker, Ilse Jonkers, Jos Vander Sloten

**Affiliations:** Department of Mechanical Engineering, KU Leuven, Celestijnenlaan 300c, Box 2419, Heverlee, 3001 Belgium; Faculty of Kinesiology and Rehabilitation Science, KU Leuven, Tervuursevest 101, Box 1500, Heverlee, 3001 Belgium

**Keywords:** In-vitro gait simulations, Ankle, Pressure distribution, ROM

## Abstract

**Background:**

Understanding the development of ankle osteoarthritis (OA) is of high importance and interest; however its causality is poorly understood and several links to joint loading conditions have been made. One way of quantifying joint loading conditions is by measuring the intra-articular pressure distribution during gait simulations performed by in-vitro experimental set-ups. However the effect of inserting a pressure sensing array in the ankle joint could potentially disturb the proper kinematics and therefore the loading conditions.

**Methods:**

In this study, we performed in-vitro gait simulations in 7 cadaveric feet, before and after inserting a pressure sensing array and quantified the effect on the joints range of motion (ROM). The gait was simulated with a stance phase duration of one second using a custom build cadaveric gait simulator (CGS).

**Results:**

The results show a limited effect in the ROM for all the joints of the hind foot, not exceeding the variability observed in specimens without a sensor. However, no consistent direction (increase/decrease) can be observed.

**Conclusion:**

The results suggest that even though the effect of inserting a pressure sensing array is minimal, it needs to be evaluated against the demands/requirements of the application.

## Background

Joint loading conditions are believed to play an instrumental role in the development and progression of osteoarthritis (OA), especially in joints of the lower limbs that are subjected to higher loads during many activities of daily living [1]. Specifically for the ankle joint (i.e. the joint between the tibia, fibula and talus), several investigators have linked the onset of OA and appearance of osteophytes to the location of high strains and stresses in the joint [2–4]. These can be caused by trauma, inducing joint instability with an abnormal and unfavourable loading pattern on the cartilage [5–7]. Quantification of the intra-articular pressure distribution in normal or post-traumatic situations is thus of high importance.

One approach for quantifying intra-articular pressure is to compute it by means of modelling, either using Finite Element Analysis (FEA) [8–10] or musculoskeletal forward or inverse dynamics simulations [11, 12]. For FEA models the specific geometry of the bones and cartilage layers is documented through computed tomography (CT) and/or magnetic resonance imaging (MRI) and three dimensional (3D) volumetric meshes are constructed in a computer environment. Material properties are assigned and motion is imposed virtually, allowing for the pressure between the cartilage layers to be calculated. The motion imposed to the joint is based either on captured in-vivo kinematics, or is chosen to represent the standard gait cycle, and most often, a simple rotation in one direction (e.g. plantar/dorsiflexion) is imposed [9, 10]. However, as these models are sensitive to changes in material properties of the modelled bodies and the kinematics imposed, they can only give a qualitative description of the pressure distribution. To be used more quantitatively, they need to be validated against direct measurements of intra-articular pressure distribution.

Pressure sensitive arrays have been used during in-vitro experimentation to measure intra-articular pressure distribution. After tissue dissection, the sensing array is inserted in the joint and captures pressure either statically [13, 14] using Fuji-Film, which is a thin film that changes in colour proportional to the load that is applied on it. To measure pressure distribution dynamically, piezo-resistive (e.g. TekScan) or capacitive (e.g. Novel) sensors should be used. These sensors translate the force applied over an area into an electrical signal and can therefore capture pressure distribution over a period of time. During dynamic measurements, the pressure distribution throughout stance-phase can be measured in an in vitro-setup [5, 15–20]. It is however important to verify whether the sensor insertion interferes with the joint kinematics, as differences in kinematics will also affect the joint loading conditions. Although the sensors used are minimal in thickness and no major dissections are performed, small changes in the configuration of the joint might have an important effect. However, so far, the effect of sensor insertion on hind foot kinematics has not yet been reported in literature.

In this study, we perform gait simulations before and after inserting a pressure array in the ankle joint of cadaveric specimens. A custom made cadaveric gait simulator (CGS), with documented high level of repeatability [21], is used. The simulations are performed under identical speed, muscle actuation pattern and imposed tibial kinematics. We hypothesise that the insertion of the pressure sensitive array does not affect the kinematics of individual bones during stance phase. To verify this hypothesis the kinematics of individual bones of the hindfoot are measured to quantify the effect of the sensor insertion on the range of motion (ROM) of hindfoot joints.

## Methods

7 freshly frozen cadaveric specimens were amputated mid-tibially and were used to perform gait simulations using a CGS that was previously validated against in vivo kinematics measured using intra-cortical pins [21]. The CGS is able to simulate stance phase on cadaveric feet specimens by imposing tibial kinematics in two translations (anterior-posterior and distal-proximal directions) and one rotation (sagittal plane) and by activating 9 muscles (Peroneal muscles, Extensor hallucis, Extensor digitorum, Tibialis anterior, Tibialis posterior, Flexor hallucis, Flexor digitorum, Gastrocnemius, Soleus) grouped in 6 groups [22] over the duration of stance phase (1 sec). The CGS is using a specimen specific model for the input tibial kinematics [23] and is operating in an inertial control loop [24], that allows performing physiologic simulations without a pre-defined trajectory for the vertical ground reaction force (vGRF) or the kinematics in the vertical direction. Intra-cortical pins (diameter: 4 mm, length: 50 mm, ICOS, New Deal, France) were inserted in 5 bones of each specimen (Tibia, Talus, Calcaneus, Naviculair, Cuboid). On top of each pin, a cluster of four active markers was mounted and its motion was captured by a Krypton Optoelectronic Motion Capture System (Krypton K 600, Metris, Belgium). The position of the markers was used to determine the 3D kinematics of the bones during gait simulations.

**Fig. 1:**
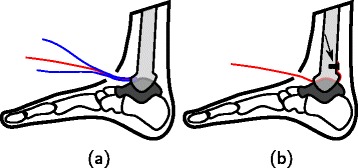
Insertion and fixation of the sensor in the ankle joint. **a** The sensor (red) is fitted between two rigid plastic surfaces (blue) and is pushed between the tibia (grey) and the talus (dark grey). **b** Once the sensing area is positioned in the center of the joint, the rigid surfaces are removed, and the sensor is fixated on the posterior side with a screw (indicated with an arrow)

For each specimen, two sets of measurements were performed: *1)* gait simulations with the specimen intact and *2)* gait simulations after inserting a pressure sensitive array in the ankle joint. For each set, 15 repetitions were performed. For the second set of measurements, the intra-articular pressure distribution in the ankle joint was measured using a Tekscan #5033 sensor (Tekscan Inc, Boston, MA). The sensor holds an array of 32×46 individual sensels in an area of 38.4×26.7 mm and has a thickness of 0.1 mm. The sensor was selected given its minimal thickness and overall dimensions, to minimize interference with normal joint function. An anterior vertical incision through the skin, inferior extensor retinaculum and joint capsule was made to access the ankle joint. Furthermore, a posterior incision was performed to give access to the posterior side of the joint. To position the sensor in the articulation, it was first positioned between two rigid plastic surfaces of the same width (Fig. 1a). The three pieces were then pushed through the joint until the sensing area was positioned in the centre of the joint and was fixated on the posterior side of the tibia using a metallic screw (Fig. 1b). The two plastic sheets where then removed. No tendons or ligaments were harmed during these incisions and their line of action was not disturbed. The two incisions were finally stitched, leaving a small opening for the side of the sensor that was connected to the reader. The operation was performed by an experienced foot surgeon.

### Data analysis

For determining the bone kinematics throughout stance phase, coordinate frames based on anatomical landmarks were constructed for each bone. The 3D bone motion was calculated by the projections of the coordinate frame of the distal bone on the planes of the proximal bone, over the duration of stance phase for each bone combination.

The ROM for each direction and bone combination was calculated as the difference between the minimal and maximal rotation over stance phase. An estimated difference in the ROM was calculated and a non-parametric test for statistical differences (Wilcoxon rank sum test) between the pre and post-sensor insertion sets was performed for each foot individually, as well as for the grouped results of all specimens. The non-parametric test was chosen as it does not assume normality of the measurements. Therefore, individual specimen response was differentiated from group results, as high variability between specimens in cadaveric testing could obscure significant individual effects. Finally, all calculations were performed for different periods of stance phase in order to detect the part of stance phase that the differences were introduced. The stance phase intervals that were analysed were from 0 to 20 %, from 21 to 80 % and from 81 to 100 %, representing the initial double support (IDS), single support (SS), and terminal double support (TDS) phases of the gait cycle. For each of these analyses, the level of significance was set to 95 % (p =.05). To determine whether the differences observed were relevant, the variability of the ROM before inserting the sensor was calculated based on all the measurements for each bone combination and plane.

## Results

**Fig. 2:**
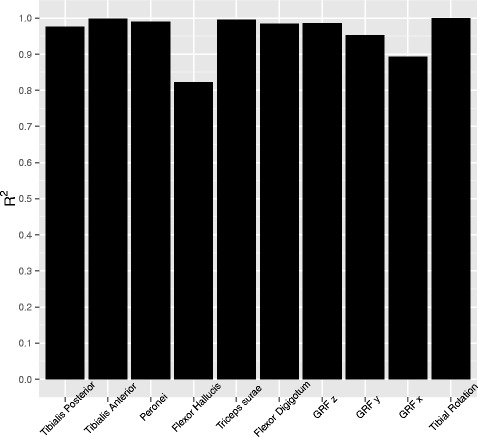
Repeatability of muscle actuation during gait simulations, as expressed by *R* ^2^ values. The forces applied before and after Tekscan insertion, the measured ground reaction forces (GRF) in three directions and the tibial rotation were compared

To demonstrate the repeatability of the imposed muscle actuation during the simulations prior and after the sensor insertion, a regression analysis of the applied forces on the tendons of the cadaver pre- and post-insertion was performed (Fig. 2). Very high repeatability (*R*^2^>0.97) is reported for all muscle actuations, except the Flexor Hallucis (*R*^2^=0.803). Also high repeatability (*R*^2^>0.9) is reported in the measured ground reaction forces in all three directions and for the tibial rotation that was imposed.

### Differences in ROM during stance phase

**Fig. 3:**
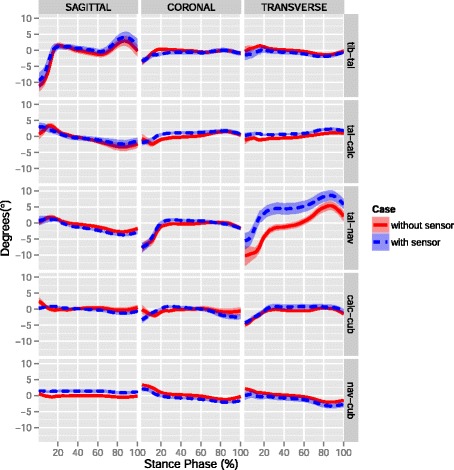
Resulting kinematics of 6 bone combinations in three directions for one specimen. The average relative rotations of the simulations without (blue) and with (red) the sensor inserted are visible

**Fig. 4:**
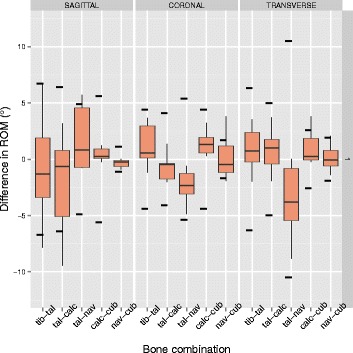
Difference in ROM for all specimens for each bone combination and direction (positive means increase). The width of each box represents the interquartile range, while the bottom and top end of the whiskers represent the lowest and highest value still within 1.5 of interquartile ranges respectively. The horizontal line inside the box represents the median. The horizontal dotted lines represent the one standard deviation of the ROM in all the measurements without the sensor

The resulting kinematics of the six bone combinations for the rotations around three anatomical planes, for the measurements before and after the sensor insertion, are presented in Fig. 3. The summary of the differences in ROM for all specimens and for each bone combination and direction is presented in Fig. 4. The greatest differences were detected for the talo-navicular in transverse and coronal planes with a 3.2 and 1.6 ° decreased ROM (Table 1). Furthermore, the talo-calcaneal joint had a 1.2 ° decreased ROM in the coronal plane. The tibio-talar joint exhibited a less than 1 ° difference in all three plane. None of these differences was statistically significant.

**Table 1:** Changes in ROM after the sensor insertion for all specimens and bone combinations

Plane		tib-tal	tal-calc	tal-nav	calc-cub	nav-cub
SAGITTAL	Specimen 1	-1.3	-0.5	-0.8	0.4	-0.1
	Specimen 2	-4.2	-0.7	0.7	0.1	-0.4
	Specimen 3	3.8	-9.5 *	-10.6 *	-0.3	0
	Specimen 4	0	-7.8 *	-0.7	-14.5 *	-0.9
	Specimen 5	6.7 *	2.1	5.7 *	1.2	-1.5 *
	Specimen 6	-7.9	-2.4	4.3	0.1	-0.1
	Specimen 7	-2.3	3.2	4.9	1.1	-0.2
	All Specimens	-0.9	-0.7	1	-0.1	-0.3
	StD	6.7	6.4	4.9	5.6	1.1
CORONAL	Specimen 1	0.1	-0.4	-0.6	0.3	-0.5
	Specimen 2	0.4	-0.7	-2.3	2	-1
	Specimen 3	-14.1 *	-10.1 *	2	1	1.4
	Specimen 4	0.9	-0.4	-1.9	-12.3 *	-1.4
	Specimen 5	3.7	1.3	-2.8	0.4	0.9
	Specimen 6	8.5 *	-2	-4.9	3.2	3.8 *
	Specimen 7	-1.2	-1.5	-3.4	1.7	-1.9 *
	All specimens	-0.9	-1.2	-1.6	0.1	-0.6
	StD	4.4	4.1	5.4	4.4	1.7
TRANSVERSE	Specimen 1	-0.2	0.6	0	0.2	-0.8
	Specimen 2	2.5	1.8	-3.5	-0.3	0.7
	Specimen 3	-16.4 *	-15.9 *	-34.3 *	0.9	-1.4
	Specimen 4	-0.3	-0.8	-4.2	-8.3 *	0.8
	Specimen 5	3.5	1.4	-4.7	3.8 *	-0.1
	Specimen 6	1.6	3.7	-1.5	0.2	-0.4
	Specimen 7	-2	-1.9	7.7	2.8 *	2 *
	All specimens	0.6	-0.1	-3.2	-0.6	-0.2
	StD	6.3	5	10.8	2.6	1.9

Statistical significant differences within the standard deviation rate are reported with a star (*, *p*<0.05)

### Changes in ROM during different phases of stance

**Fig. 5:**
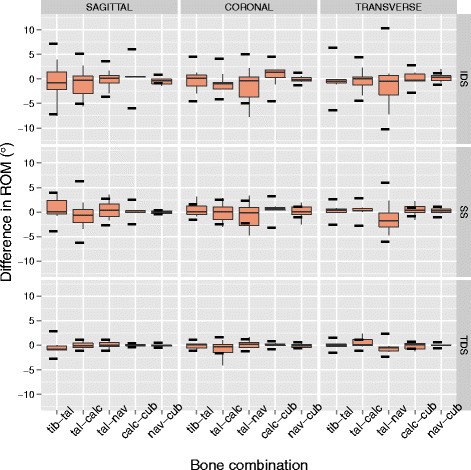
Differences in ROM pre- and post- sensor insertion, broken down into three different parts of stance phase. The top figure represents differences from 0 to 20 % of stance phase IDS, the middle from 21 to 80 % SS and the bottom from 81 to 100 % TDS

A similar response as when analysing the ROM for stance phase duration, is present for the ROM during the three different phases of stance (Fig. 5). For the IDS phase, the greatest differences appear for the talo-navicular joint in the transverse plane (1.7 ° decrease, Table 2), the tibio-talar joint in the coronal plane (1.6 ° decrease, Table 3) and the talo-calcaneal joint in the sagittal plane (1.3 ° decrease, Table 4). The differences in the SS phase are further limited to the talo-naviculair joint in the transverse plane (2.4 ° decrease) and the tibio-talar joint in the sagittal plane (1.4 ° increase). Finally, for the TDS phase the talo-navicular joint ROM decreased in the transverse plane (1.4 °) and the tibio-talar joint ROM decreased in the sagittal plane (1 °). None of these differences were statistically significant.

**Table 2:** Differences of ROM pre- and post- sensor insertion for all bone combinations for the transverse direction. The results are broken down for the three phases of stance, corresponding to the initial double support phase (0 to 20 %), single stance (21 to 79 %) and final double support phase (80 to 100 %)

Phase		tib-tal	tal-calc	tal-nav	calc-cub	nav-cub
IDS	Specimen 1	0	0.1	-0.2	0.7	-0.6
	Specimen 2	-0.4	0.5	-0.9	-0.3	0.4
	Specimen 3	-16.9 *	-14.1 *	-33.1 *	-0.5	1.9 *
	Specimen 4	-0.5	-3.3	1	-8.5 *	0.5
	Specimen 5	1.3	-0.1	-6.6	1.3	-0.7
	Specimen 6	-1	2.3	-2.4	-0.4	0
	Specimen 7	-0.9	-1.7	11.8 *	3.9 *	0.9
	All specimens	-0.2	-0.3	-1.7	-0.6	0.5
	StD	6.4	4.4	10.1	2.8	1.2
SS	Specimen 1	-0.1	0.3	0.1	-0.2	-0.2
	Specimen 2	-0.1	0.3	-2.3	0	0.9
	Specimen 3	-5.8 *	-6 *	-13.7 *	1.3 *	-0.3
	Specimen 4	0.5	2.1	-4.7	-1.6 *	0.3
	Specimen 5	2.3	0.8	2.3	2.2 *	0.9
	Specimen 6	0.8	0.1	-3.2	1 *	-0.1
	Specimen 7	0.4	0.6	-1	0.3	0.3
	All specimens	0	-0.1	-2.4	0.5	0.3
	StD	2.6	2.8	6	0.9	1.1
TDS	Specimen 1	0	0	-0.2	0.5	-0.2
	Specimen 2	1.3	2.3 *	-1.1	-0.4	0
	Specimen 3	-4 *	-3.7 *	-6.4 *	-1.1 *	0.1
	Specimen 4	0.1	1.2 *	-1.3	-1.2 *	0.6
	Specimen 5	-0.2	0.1	-0.4	0.6	0
	Specimen 6	0.3	1.1	-0.3	0.2	0
	Specimen 7	-0.3	-0.2	-0.6	0.1	0.1
	All specimens	-0.2	0.1	-1.4	-0.3	0
	StD	1.5	1.1	2.3	0.7	0.6

Statistical significant differences within the standard deviation rate are reported with a star (*, *p*<0.05)

**Table 3:** Differences of ROM pre- and post- sensor insertion for all bone combinations for the coronal direction

Phase		tib-tal	tal-calc	tal-nav	calc-cub	nav-cub
IDS	Specimen 1	-0.1	-0.5	-0.4	0	-0.5
	Specimen 2	-1.8	0.8	0.3	1.2	-0.2
	Specimen 3	-14.1 *	-10.3 *	2.2	5.8 *	0
	Specimen 4	0.3	-0.9	0.5	-13.9 *	0.5
	Specimen 5	1	-1	-2.8	-1.1	-0.4
	Specimen 6	1.3	-2.1	-7.7	1.6	2.2 *
	Specimen 7	-2.9	-1.9	-4.6	1.9	-0.6
	All specimens	-1.6	-1.2	-0.4	0.3	0.1
	StD	4.5	4.1	5	4.5	1.3
SS	Specimen 1	-0.1	0.1	-0.1	0.4	0.1
	Specimen 2	0.1	-1.3	-3 *	0.8	-0.1
	Specimen 3	-3.4 *	-1.7	-4.7 *	1.7	0.5
	Specimen 4	0.5	-3 *	-2	-8 *	-2.5 *
	Specimen 5	1.9 *	2.8 *	1.1	0.4	1.9 *
	Specimen 6	3.1 *	1	0.7	1.1	1.6 *
	Specimen 7	-0.9	1	3.4 *	0.7	-1
	All specimens	0.5	0	-0.3	0.3	-0.1
	StD	1.6	2.5	2.3	3.2	1.1
TDS	Specimen 1	0.1	-0.1	-0.4	0	-0.1
	Specimen 2	-1	-1.4	-3.8 *	0.4	-0.7 *
	Specimen 3	-3.6 *	-4.1 *	0.7	0.3	-0.2
	Specimen 4	0.3	-1.6 *	-0.5	-0.6	-0.9 *
	Specimen 5	2.1 *	0.5	0.3	0.1	0.3
	Specimen 6	-0.1	1	1.6	0.2	0.3
	Specimen 7	0.1	-0.3	0.2	-0.1	0.1
	All specimens	-0.2	-0.6	0.1	0.1	-0.1
	StD	1.1	1.6	1.2	0.8	0.6

Statistical significant differences within the standard deviation rate are reported with a star (*, *p*<0.05)

**Table 4:** Differences of ROM pre- and post- sensor insertion for all bone combinations for the sagittal direction

Phase		tib-tal	tal-calc	tal-nav	calc-cub	nav-cub
IDS	Specimen 1	-0.5	-0.9	-0.7	0.4	0.1
	Specimen 2	-7	0.3	0.1	0	-0.1
	Specimen 3	3.9	-5.5 *	-2.9	0.6	-1.4 *
	Specimen 4	-1.1	-12.9 *	-0.9	-15.2 *	-1.3 *
	Specimen 5	2.1	0.6	0.6	0.3	-0.7
	Specimen 6	-18.5	-3.6	0.8	0.5	-0.4
	Specimen 7	-2.5	2.7	1.7	1.6	0.1
	All specimens	-1.2	-1.3	0.4	0.3	-0.6
	StD	7.2	5.1	3.6	6	0.9
SS	Specimen 1	-0.2	0.3	-0.2	-0.1	-0.1
	Specimen 2	-0.8	-0.8	0.3	-0.1	-0.3
	Specimen 3	1.5	-3.4	-5.6 *	0.3	1 *
	Specimen 4	-0.5	-8.6 *	-1.7	-6.5 *	0.4
	Specimen 5	4.1 *	1.9	3.6 *	0.4	-0.5 *
	Specimen 6	3.3	0.7	1.8	0.2	0
	Specimen 7	0.1	-0.7	1.4	0.2	-0.3
	All specimens	1.4	-0.3	0.3	-0.2	0
	StD	3.9	6.3	2.7	2.5	0.4
TDS	Specimen 1	-0.9	-0.1	0	-0.2	-0.1
	Specimen 2	0.4	-0.7	0.7	0.2	-0.4
	Specimen 3	-4 *	-2.1 *	-1.3 *	0.1	0.1
	Specimen 4	-0.3	2.9 *	-0.1	-0.9 *	0
	Specimen 5	1.3	-0.1	0.9	0.5 *	-0.3
	Specimen 6	-0.9	1	0.3	0.2	0
	Specimen 7	-0.8	-0.4	-0.3	-0.1	0.1
	All specimens	-1	-0.1	0.2	-0.1	-0.1
	StD	2.8	1.1	1.1	0.4	0.5

Statistical significant differences that were observed are reported with a star (*, *p*<.05)

## Discussion

In this study we investigate the effect of the insertion of a pressure sensing array in the ankle joint, by quantifying the difference in ROM in cadaveric specimens during simulated gait. Several gait cycles were performed using freshly frozen cadaveric specimens, and the cycles were performed prior and after the insertion of a pressure sensing array. The ROM of different joints was calculated and compared statistically between the two situations. Finally, the input forces applied on the specimens during the measurements were compared between the two sets of measurements in order to showcase the similarity of the boundary conditions. The comparison of the forces suggests that the conditions after inserting the sensor were identical to those before, as high values of correlation appear (*R*^2^>0.97 for all muscles, except for flexor hallucis *R*^2^=0.8 and *R*^2^>0.92 for all directions of the ground reaction force). Therefore the differences found in ROM can be assumed to be affected only by the sensor insertion. The lowest value of correlation for the flexor hallucis muscle relates to the fact that this muscle has the least actuation during stance phase (max F=8.5 N) and thus the noise-to-signal ratio of the measured force is higher. However, the combination of a lower contribution of the muscle in the gait and the still relatively high *R*^2^ value does not contaminate the repeatability of the measurements.

The results of the differences in ROM demonstrate a median difference in ROM after the sensor insertion less than 2.5 °, for all bone combinations and directions. The largest differences occurred in the tibio-talar and talo-navicular joints in sagittal plane rotation, and in talo-navicular joint in transverse plane. However, these differences were within the variability observed in the specimens without the sensor and were not detected to be statistically significant. The individual specimens demonstrated only limited significant differences, with the exception of specimen 3, that demonstrated significant differences in many joints and directions, both for the whole stance phase duration but also for the specific phases of stance.

Even though larger changes in ROM were expected in the ankle joint, where the incision was made, the largest differences appear in the talo-naviculair joint. This finding can be explained by the fact that this joint is less supported, especially during the initial phase of stance. This seems to be confirmed by the larger differences in ROM at the beginning than in the middle and end of stance phase. Such results agree with previous findings by Okita et al. [25], who observed higher compliance of the midtarsal joints during foot flat and push-off phases, compared to the loading response phase.

This study is the first one to quantify the effect of a sensing array insertion in the ankle joint. Several researches have reported findings on intra-articular pressure in the ankle joint [9, 15] or validated finite element models with it [8]. However, even in the absence of significant and relevant differences in the ROM after sensor insertion, it is important to notice that, there is some variability between different specimens, with some specimens presenting increase and others decrease in ROM. This variability shows that even with a repeatable experimental protocol, it is difficult to predict the effect of inserting a sensor in the ankle joint. Therefore attention is needed when interpreting intra-articular pressure measurements obtained from in-vitro simulations, as such measurements might not necessarily reflect to the pre-sensor insertion situation. An example of this can be seen for specimen 3, where much higher difference in ROM is observed, probably due to slightly different incision during the insertion of the sensor. When such a difference is detected, the results of the intra-articular pressure distribution cannot be considered reliable, and the measurements from such specimens should be discarded. Finally, when using the experimental pressure measurements for model validation, a margin of error of 2.5 ° on the associated kinematics should be taken into account, and a sensitivity analysis should be performed to judge if such an error margin would induce relevant differences for the envisaged application.

## Conclusion

This study investigated the influence of inserting a pressure sensing array in the ankle joint on the kinematics of the hindfoot bones, during simulated roll-offs. The influence was determined by the difference in ROM of five joints during pre- and post-insertion simulations. The limited differences in ROM indicate that the kinematics are not affected significantly by such a procedure and therefore the measurements obtained can be considered representative of the pre-insertion conditions.
